# Self-Perception of Changes in Routines in Adults and Older Adults Associated to Social Distancing Due to COVID-19—A Study in São Paulo, Brazil

**DOI:** 10.3389/fpsyg.2021.607559

**Published:** 2021-02-23

**Authors:** Adriana Machado-Lima, Angélica Castilho Alonso, Débora Gozzo, Gisele Garcia Zanca, Guilherme Carlos Brech, José Maria Montiel, Marta Ferreira Bastos, Priscila Larcher Longo, Sandra Regina Mota-Ortiz

**Affiliations:** Postgraduate Program in Aging Sciences, São Judas Tadeu University, São Paulo, Brazil

**Keywords:** adults, aged, COVID-19, perception, social distance

## Abstract

COVID-19 is an acute respiratory illness with higher mortality in older adults. This condition is spread person-to-person through close contact, and among policies employed to decrease transmission are the improvement of hygiene habits and physical distancing. Although social distancing has been recognized as the best way to prevent the transmission, there are concerns that it may promote increased depression symptoms risk and anxiety, mainly in older adults. This cross-sectional study aimed to verify self-concept of social distancing in adults compared to older adults. All participants, over 18 years and residents of São Paulo state (Brazil), were invited to join this research study by a message application and answered an interdisciplinary questionnaire during the period from May 23 to June 23, 2020. The questions were divided into the following aspects: sociodemographic data, financial conditions, routine-related perception, perception of health, physical and emotional state, and eating habits. The younger adult group was composed of 139 participants, with a mean age of 43.15 years (±10.92), and the older adult group was composed of 437 participants with a mean age of 67.59 years (±6.13) of both sex. Changes in routine during the period of social distance were reported by 95% of adults and 96.8% of older adults, but adults indicated more significant alterations in routine. Although there was no difference between groups for several aspects, adults revealed greater alterations in sleep quality, evacuation frequency, and more difficulty to perform daily activities at home. Further studies are necessary to follow up the impacts of social distancing among adults and older adults in different socioeconomic contexts to better understand the long-term alterations and the necessity of interventions.

## Introduction

The aging process is complex and multifactorial, related to morphological, physiological, biochemical, social, and psychological decline (Carmona and Michan, 2016), including the increased risk of infections and decrease of immunological mechanisms (Pawelec, 2018). In addition, older adults have multiple comorbidities, which increase the chance of hospitalizations and may be considered an additional complicating factor in this pandemic moment (Shahid et al., 2020). Taken together, these facts could explain the higher severity of COVID-19 in the older adult population (Greve et al., 2020). COVID-19 is an acute respiratory illness caused by Severe Acute Respiratory Syndrome Coronavirus 2 (SARS-CoV-2), an enveloped, positive single-stranded large RNA virus that infects humans, but also a wide range of animals (Velavan and Meyer, 2020).

In Brazil, the absence of effective health public politics by the Federal Government became more difficult to combat COVID-19 (Lancet, 2020), which promoted high rates of infected people and deaths. According to official data (Sistema Único de Saúde, 2020) since March 2019, Brazil ranks among the countries with the highest numbers of infected people and deaths caused by COVID-19. At this scenario, São Paulo, the largest State in the country accounts for the greatest cases reported (Prefeitura de São Paulo, 2020).

As COVID-19 is spread person-to-person through close contact, by droplets and aerosols (Jayaweera et al., 2020; Perisetti et al., 2020a), several policies have been employed to decrease transmission, such as improvement of hygiene habits, mask wearing, and in most countries, physical distancing recommendations (Chu et al., 2020; Perisetti et al., 2020a). Although social distancing is recognized as the best way to prevent the contamination, there are concerns that it may promote the increased depression symptoms risk and anxiety in older adults (Armitage and Nellums, 2020; Castelnuovo et al., 2020; Santini et al., 2020).

For the aged, the impact of physical distancing could be worse because they have less online social interaction (Berg-Weger and Morley, 2020). Moreover, it has been widely proposed that the maintenance of daily activities are fundamental to keep the quality of life and physical and mental health of older adults (Manini et al., 2006; Britto et al., 2018; Chen et al., 2020; Hammami et al., 2020). Therefore, the present study aimed to evaluate the perception of the impacts of social distancing among younger adults and older adults in Brazil using an interdisciplinary questionnaire.

## Materials and Methods

### Participants

This is a cross-sectional study approved by the Ethics Committee (Universidade São Judas Tadeu), No. 4.067.240.

The invitation was performed by a multiplayer app for instant messaging for smartphones (WhatsApp). If interested, the probable participant received the consent terms and questionnaire by Google Forms link. Inclusion criteria were 18 years or older, able to read and respond to the online questionnaire, and a resident of São Paulo State (Brazil). The invitation letter was restricted to people living in São Paulo; however, in case of response from people living outside São Paulo, they were not included. In addition, exclusion criterion was not completing the questionnaire. All invited participants fulfilled the inclusion criteria, during the period from May 23 to June 23, 2020, since social distancing was instituted by the state government on March 22, 2020.

### Procedures

An interdisciplinary questionnaire was developed specifically to this study, to evaluate the cross-section of the moment to this population using *Google Forms* tool (Google LLC, CA, United States). The questions were divided into the following aspects: sociodemographic data, financial conditions, perceptions of routine, health, physical and emotional status, and eating habits regarding the recall and perception, respectively, of the periods before and during social distancing. All participants had access to the instrument after virtually signing the informed consent form.

### Statistical Analyses

Data related to perception before and during the social distancing were compared between adult (18–59 years) and older adult (≥60 years) groups, and a categorical chi-square test was applied (Statistical Package for the Social Sciences^®^ SPSS software, version 25, IBM, NY, United States). Perception of sleep quality, practice of physical activity, and tiredness sensation before and after the beginning of social distancing were compared using the Wilcoxon test for intragroup analysis and Mann–Whitney *U* test for intergroup analysis (GraphPad Prism^®^ 8.0, GraphPad Software Inc., CA, United States). The significance level for all statistical tests was established at 5%.

## Results

All questions have been answered, since all the questions were mandatory to submit the forms. There was no missing data.

The adult group (20–59 years) was composed of 139 participants and the older adult (60–98 years) group was composed of 437 participants of both sex. Changes in routine during the period of social distance were reported by 95% of adults and 96.8% of older adults. Demographic characteristics of adults and older adults are presented at Table 1.

**TABLE 1:** Sociodemographic characteristics of adults (*n* = 139) and older adults (*n* = 437) included in the present study.

	Adults (%)	Older adults (%)	χ^2^ (*p*)
**Age (mean ± standard deviation years)**			
	43.15 ± 10.92	67.59 ± 6.13	Does not apply
**Sex**			
Male	31 (22.3)	137 (31.4)	4.179
Female	108 (77.7)	300 (68.6)	(0.041)*
**Years of study**			
1–4	2 (1.4)	34(7.8)	16.100 (≤0.001)*
5–8	2 (1.4)	32 (7.3)	
9–11	22 (15.8)	77 (17.6)	
>12	113(81.3)	294 (67.3)	
**Marital status**			
Single	40 (28.8)	40 (9.2)	43.806 (≤0.001)*
Married	74 (53.2)	259 (59.3)	
Widowed	4 (2.9)	69 (15.8)	
Divorced/Separated	21 (15.1)	69 (15.8)	
**Number of people living with you**			
0	16 (11.5)	77 (17.6)	40.078 (≤0.001)*
1	27 (19.4)	161 (36.8)	
2	31 (22.3)	110 (25.2)	
>3	65 (46.8)	89 (20.4)	
**Brazil minimum monthly wage^#^**			
1–3	25 (18.0)	118 (27.0)	3.652 (0.161)
4–6	45 (32.4)	116 (26.5)	
>6	69 (49.6)	203 (46.5)	

*Represents statistical difference between the groups by chi-square test. ^#^1 Brazil minimum monthly wage = 197.71 USD. https://www.bcb.gov.br/en#!/n/EXCHANGERATES (accessed on September 13, 2020).

Adults reported greater income impairment (*p* = 0.002) and received more financial assistance from the government due to the pandemic (*p* = 0.006) than older adults. Most older adults were at least 15 days away from meeting relatives or friends who did not live with them (*p* = 0.025). Older adults reported higher time of remaining at home since the beginning of social distancing than adults (*p* < 0.0001) (Table 2).

**TABLE 2:** Perception of financial conditions and social distancing-related aspects of adults (*n* = 139) and older adults (*n* = 437).

	Adults *n* (%)	Older adults *n* (%)	χ^2^ (*p*)
**Did you have compromised income due to the pandemic?**			
There was no compromise	58 (41.7)	243 (55.6)	17.479 (0.002)*
Decreased less than 50%	31 (22.3)	111 (25.4)	
Decreased 50%	19 (13.7)	31 (7.1)	
Decreased more than 50%	20 (14.4)	34 (7.8)	
There was no income	11 (7.9)	18 (4.1)	
**Do you have any financial assistance during the pandemic?^#^**			
Family members	12 (8.8)	35 (8.0)	0.078 (0.450)
Extra services	27 (5.1)	17 (3.9)	0.387 (0.341)
Savings	37 (27.0)	104 (23.9)	0.559 (0.261)
Financial loan	1 (0.7)	8 (1.8)	0.819 (0.326)
Government aid	14 (10.2)	17 (3.9)	8.177 (0.006)*
**How many days have you not met face to face relatives or friends who don’t live in your house?**			
0	6 (4.3)	13 (3.0)	9.356 (0.025)*
1–7	27 (19.4)	46 (10.6)	
8–15	14 (10.1)	37 (8.5)	
>15	92 (62.2)	341 (78.0)	
**Have you received support from your relatives?**			
No—1	19 (13.7)	70 (16.1)	4.364 (0.359)
2	13 (9.4)	23 (5.3)	
3	22 (15.8)	55 (12.6)	
4	19 (13.7)	66 (15.2)	
Very—5	66 (47.5)	223 (50.8)	
**When was the last time you went out?**			
Did not go out	4 (2.9)	94 (21.5)	32.472 (≤0.001)
1–7 days	113 (81.3)	251 (57.4)	
>8 days	22 (15.8)	92 (21.1)	

*Represents statistical difference between the groups by chi-square test. ^#^More than one answer was allowed for these variables.

Data of health perception and physical status are shown in Table 3. Adults had greater changes related to stool frequency when compared to older adults (*p* < 0.0001). Adults referred to greater difficulties while most older adults reported no difficulties in carrying out their activities at home (*p* < 0.0001).

**TABLE 3:** Perception of health and physical state of adults (*n* = 139) and older adults (*n* = 437) during social distancing due to the COVID-19 pandemic.

	Adults *n* (%)	Older adults *n* (%)	χ^2^(*p*)
**How would you rank your health?**			
Really bad—1	0	1 (0.2)	8.457 (0.133)
2	1 (0.7)	2 (0.5)	
3	4 (2.9)	6 (1.4)	
4	49 (35.5)	180 (41.2)	
Excellent—5	57 (40.6)	196 (44.9)	
**Are you more concerned with your hygiene habits?**			
No, nothing—1	3 (2.2)	20 (4.6)	1.677 (0.795)
2	1 (0.7)	3 (0.7)	
3	9 (6.5)	30 (6.9)	
4	23 (16.5)	72 (16.5)	
Yes, a lot—5	103 (74.1)	312 (71.4)	
**Did social distancing change your stool frequency?**			
Yes, decreased	22 (15.8)	31 (7.1)	
No	93 (66.9)	370 (84.7)	21.132 (≤0.001)*
Yes, increased	24 (17.3)	36 (8.2)	
**Do you have difficulties carrying out your daily activities at home?**			
No, nothing—1	47 (33.8)	225 (51.7)	52.730 (≤0.001)*
2	18 (12.9)	84 (19.3)	
3	31 (22.3)	77 (17.7)	
4	17 (12.2)	37 (8.5)	
Yes, a lot—5	26 (18.7)	12 (2.8)	
**Did you suffer from any kind of fall in this period of social distancing?**			
No	130 (93.5)	413 (95.2)	0.568 (0.451)
Yes	9 (6.5)	21 (4.8)	

*Represents statistical significance between groups (p ≤ 0.05).

**FIGURE 1:**
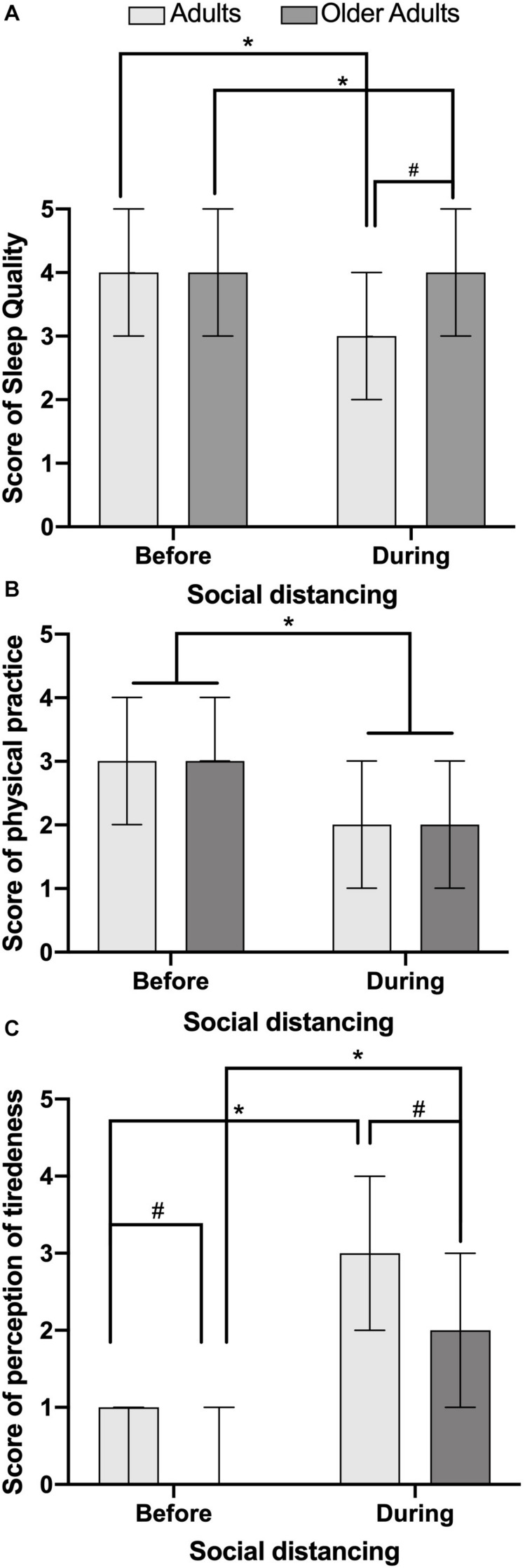
Recall and perception of sleep quality **(A)**, physical practice **(B)** and tiredness level **(C)** for adults and older adults residents in São Paulo, respectively of periods before and during the social distancing due to COVID-19. ^∗^Represents statistical differences intragroup before and during the social distancing by Wilcoxon test (*p* < 0.05). ^#^Represents statistical difference intergroups, before and during the social distancing due COVID 19 pandemic by *U* Mann-Whitney test (*p* < 0.05).

When asked about sleep quality, adults and older adults reported a good quality of sleep before social distancing, and no statistical difference was detected between these groups (*p* = 0.959). Intragroup analysis showed that both adults and older adults pointed out an impairment in sleep quality when compared before and during the social distancing imposed by COVID-19 (Figure 1A, *p* < 0.0001 for adult and older adult comparisons). In addition, for adults, there was a worsening in sleep quality in comparison to all older adult groups during social distancing caused by the COVID-19 pandemic (Figure 1A, *p* < 0.0001 for adult and older adult comparisons). The confidence level of the median was 95.02% for adults before and during social distancing, while the confidence level was 95.05 and 95.56% for aged before and during social distancing, respectively.

Adults and older adults showed good levels of physical activities before and during social distancing, without significant difference between the moment before (*p* = 0.743) and during (*p* = 0.060) social distancing caused by the COVID-19 pandemic (Figure 1B). However, both groups showed significant reduction of physical activities after the beginning of social distancing (Figure 1B, adults *p* < 0.0001 and older adults *p* < 0.0001). The confidence level of median to adults was 95.86 and 95.02%, and for the aged, the actual confidence level was 95.56 and 95.05% before and during social distancing, respectively.

Older adults and adults had different levels of tiredness before and during social distancing (Figure 1C, both *p* < 0.0001). Both groups related significantly increased levels of tiredness during the social distance period (Figure 1C, *p* < 0.001), although perception of tiredness in adults has remained higher than in the older adults (Figure 1C, *p* < 0.0001). The confidence level of median was 95.86% for adults before and during and social distancing, while for the aged, the confidence level was 95.56 and 95.61% before and during social distancing, respectively.

There was no difference in relation to sun exposition between groups, and most subjects of both groups, adults (65.5%) and older adults (62.7%), related a sun daily exposition around 1 to 3 h while one third of the adults (33.1%) and older adults (33.4) were not exposed to the sun (*p* = 0.549).

Moreover, older adults consumed fruits (*p* < 0.0001) and vegetables (*p* = 0.046) more frequently when compared to adults, who had higher frequency of protein ingestion (*p* < 0.0001) (Table 4).

**TABLE 4:** Dietary habits of adults (*n* = 139) and older adults (*n* = 437) during social distancing due to the COVID-19 pandemic.

	Adults *n* (%)	Older adults *n* (%)	χ^2^ (*p*)
**How many fruits or fruit juice did you consume per day?**			
None	9 (6.5)	14 (3.2)	28.715 (≤0.001)*
1	56 (40.3)	91 (20.8)	
2	40 (28.8)	159 (36.4)	
3	23 (16.5)	112 (25.6)	
4	8 (5.8)	29 (6.6)	
≥5	3 (2.2)	32 (7.3)	
**How many vegetables did you consume per day?**			
None	9 (6.5)	10 (2.3)	11.300 (0.046)*
1	53 (37.7)	148 (33.9)	
2	50 (36.2)	213 (48.7)	
3	12 (8.7)	27 (6.2)	
4	6 (4.3)	18 (4.1)	
≥5	9 (6.5)	21 (4.8)	
**How much meat, chicken, fish, or egg did you consume per day?**			
None	6 (4.3)	8 (1.8)	20.255 (≤0.001)*
1	41 (29.5)	187 (42.9)	
2	61 (43.9)	178 (40.8)	
3	11 (7.9)	29 (6.7)	
4	2 (1.4)	14 (3.2)	
≥5	18 (12.9)	20 (4.6)	
**How much milk, cheese, or yogurt did you consume per day?**			
None	7 (5.0)	37 (8.5)	4.949 (0.422)
1	53 (38.1)	158 (36.2)	
2	44 (31.7)	158 (36.2)	
3	15 (10.8)	43 (9.8)	
4	9 (6.5)	22 (5.0)	
≥5	11 (7.93)	22 (5.0)	

*Represents statistical significance between groups (p ≤ 0.05).

Regarding the perception of emotional status, adults and older adults indicated a similar mood during social distancing. Most participants of both groups recorded intermediate levels between discouraged/excited and sad/cheerful. However, adults felt more insecure (*p* = 0.003), while older adults felt more cautious (*p* = 0.004). Moreover, adults missed traveling (*p* = 0.005) while older adults missed walking, going wherever they wanted (*p* = 0.038), and going out (*p* = 0.005) (Table 5).

**TABLE 5:** Perception of the emotional status for adults (*n* = 139) and older adults (*n* = 437) during social distancing due to the COVID-19 pandemic.

	Adults *n* (%)	Older adults *n* (%)	χ^2^ (*p*)
**What is the closest point of your feeling in the last 15 days?**			
Discouraged—1	15 (10.9)	25 (5.8)	9.016 (0.061)
2	29 (21.2)	65 (15.0)	
3	59 (43.1)	202 (46.7)	
4	25 (18.2)	96 (22.2)	
Excited—5	9 (6.6)	45 (10.4)	
**What is the closest point of your feeling in the last 15 days?**			
Sad—1	8 (5.8)	20 (4.6)	7.650 (0.150)
2	26 (18.7)	65 (15)	
3	74 (53.2)	201 (46.3)	
4	20 (14.4)	108 (24.9)	
Cheerful—5	11 (7.9)	40 (9.2)	
**Did spirituality help you at this time?**			
Never—1	7 (5)	32 (7.40)	7.772 (0.100)
2	8 (5.8)	25 (5.8)	
3	29 (20.9)	51 (11.8)	
4	24 (17.3)	84 (19.4)	
Frequently—5	71 (51.1)	242 (55.8)	
**Which items most explain what you are experiencing right now?^#^**			
Insecure	70 (50.4)	158 (36.2)	8897 (0.003)*
Cautious	56 (40.3)	237 (54.2)	8206 (0.004)*
Hopeful	55 (39.6)	208 (47.6)	2740 (0.098)
Boring	51 (36.70)	139 (31.8)	1137 (0.286)
**What are you missing with social distancing?^#^**			
Traveling	96 (69.1)	343 (59.5)	6.888 (0.005)*
Walking and going wherever	95 (68.3)	337 (77.1)	4327 (0.038)*
Going out	82 (59)	287 (65.7)	6888 (0.005)*
Being with my family	96 (69.1)	329 (75.3)	2110 (0.146)

*Represents statistical significance between groups (p ≤ 0.05). ^#^These variables permitted more than one answer.

## Discussion

The results showed a perception of routine alterations independent of age, and both groups reported a decrease in physical activity level and sleep quality as well as an increased tiredness perception during social distancing when compared to their reported previous status.

The decrease in physical activity level was an expected consequence of the “stay at home” recommendations. Lippi et al. (2020) reported that this decrease is greater for older adults, since it is well-known that regular physical activity is essential to maintain and/or improve muscle strength, gait, and postural balance, influencing functional independence, quality of life (Liu-Ambrose et al., 2019; Greve et al., 2020), and falls prevention (Pelicioni and Lord, 2020). Although the number of falls could not be determined, it should be considered that the mean age of the older adults in the present study was relatively low. Furthermore, data collection was performed after a period of 30–60 days of social distancing and falls report depended on participants’ memory and comprehension of what should be considered as a falling event.

Although sleep quality is also influenced by physical activity (Gothe et al., 2019), only adults reported sleep impairment during social distancing. It has been shown that adults increased screen time exposure during social distancing due to work and study demands and to keep informed (Majumdar et al., 2020). Screen blue light exposure may negatively impact sleeping due to the suppression of melatonin production (Calvo-Sanz and Tapia-Ayuga, 2020). Moreover, adults probably had more activities outside their homes and used to stay less at home before social distancing than older adults, which may be related to the reported changes in physical activity level. Poorer sleep quality may also be related to the greater difficulty to perform activities of daily living compared to older adults and the fact that the financial impact of pandemic was greater for adults, as they reported in the present study. Economical instability, income decrease, and unemployment concerns may have influenced sleep quality among adults (International Labour Organization, 2020).

There is also a great concern about the impact of social distancing on loneliness, mainly among older adults, due to its association with overall functionality decline and depressive and anxiety symptoms (Meng et al., 2020; Monahan et al., 2020; Tyrrell and Williams, 2020). According to our results, older adults usually live alone or with fewer people and have been reported to stay at home for more consecutive days and to spend longer periods without meeting people who do not live with them. Older adults also reported more frequently to miss meeting relatives, when asked about what they miss more with social distancing. However, these aspects seem to have no influence on their mood. Surprisingly, no difference was found for mood between older adults and younger adults, with both groups presenting an intermediate status from “sad” to “happy” and from “discouraged” to “excited,” suggesting that social distancing was not related to noteworthy emotional alterations between the groups. This result may be due to the relatively short period of social distancing at the time of data collection; therefore, further longitudinal studies on these aspects are necessary (Montemurro, 2020; Wang et al., 2020). In agreement with the literature, we also suggest that older adults should be followed up by health professionals in order to early identify conditions that require intervention (Fessell and Cherniss, 2020; Lades et al., 2020).

As related before, adults felt more insecure and older adults felt more cautious, which may be explained by the fact that older adults present higher risk of COVID-19 complications (Shahid et al., 2020) and, therefore, caution has been emphasized for them. Regarding the question of what people miss with social distancing, adults reported more frequently to miss traveling (Chudyk et al., 2015). In general, adults continued working at home, so traveling could bring possibilities of enjoying diverse experiences and leisure activities. Older adults reported to miss the possibility of going out. The possibility of deciding when going out, regardless of the activity involved, is related to autonomy and functionality (Armitage and Nellums, 2020).

With social distancing, people are modifying their social bonds, and this could result in a negative impact on the eating habits of the participants, especially the older adults (Allès et al., 2019). There is a correlation between social bonds and eating habits (Campos et al., 2000; Silveira et al., 2015). However, with stress, there may be changes in the quantity and the quality of the food consumed, a decrease in appetite (Petrowski et al., 2014; Reichenberger et al., 2018), as well as an increase in high caloric density food consumption. These alterations may lead to changes in glycemia, lipid profile, and consequently increased risk for the development of chronic diseases (Evers et al., 2010; Van Strien et al., 2012; Sinha, 2018). In the present study, adults reported lower frequency of fruit and vegetable intake and higher frequency of protein intake in comparison to older adults. Sidor and Rzymski (2020) have shown a decrease in fruit and vegetable consumption and a greater tendency to consume meat during social distancing among adults. Older adults usually eat less proteins, fruits, and vegetables, which may be related to the presence of chronic disease or with oral cavity alterations (Gaspareto et al., 2017; Ibge, 2019), reflecting on implications in muscle mass, such as sarcopenia and other adverse outcomes (do Nascimento Ferreira et al., 2017).

Adults reported irregularity in stool frequency, some with increased and some with decreased frequency, and this may be related to possible irregular dietary habits, lesser physical activity, and changes in sleep quality. It is important to note that microbial gut composition may be affected by stool frequency (Kwon et al., 2019), and this composition is related to health and many diseases such as obesity, diabetes (Pascale et al., 2019), and neurodegenerative diseases (Roy Sarkar and Banerjee, 2019), indicating that social distancing may be related to gut dysbiosis.

Furthermore, gastrointestinal manifestations have been increasingly recognized in patients with COVID-19 (Aziz et al., 2020; Kopel et al., 2020; Perisetti et al., 2020c). There is a silent transmission among the community starting with gastrointestinal infection, leading to changes in bowel pattern in a number of individuals (Perisetti et al., 2020b). Whereas, the incidence of COVID-19 is high in Brazil (Lancet, 2020; Sistema Único de Saúde, 2020), it should be considered that there is a chance that some participants in our sample presented asymptomatic or mild infection. However, this chance is probably small, since the majority of participants reported to observe social distancing for more than 15 days at the time of data collection.

Despite the impairments reported in physical activity and tiredness during social distancing, the majority of both groups presented a very good or excellent self-rated health status. A longitudinal study has assessed Swedish older adults and observed that self-rated health status increased during the COVID-19 pandemic. The authors have suggested that this finding could be related to the “contrast effect,” i.e., people consider their health satisfactory when compared to the potential negative effects of COVID-19 (Kivi et al., 2020).

In the present study, participants lived in São Paulo (SP-Brazil) and presented a high level of education (12 years) when compared to the average in Brazil (9.3 years) (PNAD, IBGE, 2018). Also, reported income was higher than the average in the country (1 minimum monthly wage/per person) (Ibge, 2019). These aspects may have contributed to minimize the impacts of social distancing in this sample. Furthermore, participants were recruited online; i.e., only people with internet access and digital literacy to respond to an online questionnaire were included. São Paulo is the biggest city in Brazil and has the highest economy and the highest Human Development Index of the country (0.5) (Duarte, 2020). Although the findings should not be generalized to the general Brazilian population, they represent differences between adult and older adult perceptions that are probably more related to social distancing itself than to its economical consequences.

## Limitation

Although there are relevant findings in the present study, some limitations should be highlighted. Data were subject to participant recall, which may have potential influence on results, mainly on the comparisons between pre- vs. during social distancing periods. There is also possible selection bias, since participants might respond in clusters. Furthermore, these results are specific to the urban population of São Paulo, Brazil, and although we have not included participants who declared living in other states, that information also depended on participant reports. Other sample characteristics were not assessed, such as comorbidities, COVID-19 infection, emotional conditions, employment status, and type of diet consumption before social distancing. Finally, this is a cross-sectional study and, therefore, the results must be interpreted with caution.

## Final Considerations

There are great concerns regarding the impact of social distancing on health, mainly among older adults, considering that aging is related to biopsychosocial decline. Therefore, we have hypothesized that older adults would present greater health and behavioral alterations than adults. However, while there was no difference between groups for several aspects, adults have presented a perception of greater alterations in others, such as poorer sleep quality, alterations in evacuation frequency, and more difficulty to perform daily activities at home. It is possible that adults presented more significant alterations in routine, despite the great perception of alterations reported by both groups. The reasons for our findings were related to some aspects, but alternative explanations may exist for these findings as well. Further studies are necessary to follow up the impact of social distancing among adults and older adults in different socioeconomic contexts and to better understand the long-term alterations and the necessity of interventions. Finally, it should be highlighted that, besides the impact in several aspects, social distancing is essential to slow the spread of COVID-19 and to save lives (Courtemanche et al., 2020).

## Data Availability Statement

The raw data supporting the conclusions of this article will be made available by the authors, without undue reservation, to any qualified researcher.

## Ethics Statement

This is a cross sectional study approved by the Ethics Committee (Universidade São Judas Tadeu), No. 4.067.240. The patients/participants provided their written informed consent to participate in this study.

## Author Contributions

AM-L, PL, and SM-O: writing-review and editing. AA and MB: formal analysis. DG, GZ, GB, and JM: investigation and writing-original draft. All authors contributed to the article and approved the submitted version.

## Conflict of Interest

The authors declare that the research was conducted in the absence of any commercial or financial relationships that could be construed as a potential conflict of interest.
