# Corrigendum: Self-Perception of Changes in Routines in Adults and Older Adults Associated to Social Distancing Due to COVID-19—A Study in São Paulo, Brazil

**DOI:** 10.3389/fpsyg.2021.684729

**Published:** 2021-05-19

**Authors:** Adriana Machado-Lima, Angélica Castilho Alonso, Débora Gozzo, Gisele Garcia Zanca, Guilherme Carlos Brech, José Maria Montiel, Marta Ferreira Bastos, Priscila Larcher Longo, Sandra Regina Mota-Ortiz

**Affiliations:** Postgraduate Program in Aging Sciences, São Judas Tadeu University, São Paulo, Brazil

**Keywords:** adults, aged, COVID-19, perception, social distance

In the original article, there was an error. A citation was inserted incorrectly at the end of a paragraph.

A correction has been made to *Discussion, Paragraph 6*. The corrected paragraph is shown below.

With social distancing, people are modifying their social bonds, and this could result in a negative impact on the eating habits of the participants, especially the older adults (Allès et al., 2019). There is a correlation between social bonds and eating habits (Campos et al., 2000; Silveira et al., 2015). However, with stress, there may be changes in the quantity and the quality of the food consumed, a decrease in appetite (Petrowski et al., 2014; Reichenberger et al., 2018), as well as an increase in high caloric density food consumption. These alterations may lead to changes in glycemia, lipid profile, and consequently increased risk for the development of chronic diseases (Evers et al., 2010; Van Strien et al., 2012; Sinha, 2018). In the present study, adults reported lower frequency of fruit and vegetable intake and higher frequency of protein intake in comparison to older adults. Sidor and Rzymski (2020) have shown a decrease in fruit and vegetable consumption and a greater tendency to consume meat during social distancing among adults. Older adults usually eat less proteins, fruits, and vegetables, which may be related to the presence of chronic disease or with oral cavity alterations (Gaspareto et al., 2017; Ibge, 2019), reflecting on implications in muscle mass, such as sarcopenia and other adverse outcomes (do Nascimento Ferreira et al., 2017).

The authors apologize for this error and state that this does not change the scientific conclusions of the article in any way. The original article has been updated.
