# Supplementary material for: Self-Perception of Changes in Routines in Adults and Older Adults Associated to Social Distancing Due to COVID-19—A Study in São Paulo, Brazil
Source: Front Psychol. 2021 Feb 23;12:607559. doi: 10.3389/fpsyg.2021.607559 (PMC7940366; doi:10.3389/fpsyg.2021.607559)
Supplement: Supplementary file 1 [file Data_Sheet_1.PDF]

Self-perception of changes in routines in adults and older adults associated to social distancing due to COVID-19 - a study in São Paulo, Brazil

This questionnaire is related to a research study on the impacts of social distancing recommendations due to COVID-19 on adults and older adults routine.

*Obrigatório

1. Date: *

____

Exemplo: 7 de janeiro de 2019

2. Name: *

____

3. CPF* : *

(*note: Brazilian individual taxpayer registry identification)

____

4. How old are you? *

____

5. Schooling *

Marcar apenas uma oval.

- Never studied in formal school
- Elementary school or incomplete elementary school
- First degree or complete elementary school
- High school or incomplete high school
- High school or high school
- Incomplete higher education
- Complete Higher Education

6. Marital status: *

Marcar apenas uma oval.

- Single
- Married / lives with a partner
- Widowed
- Divorced or Separated

7. What city do you live? *

____

8. What state do you live? *

____

9. How many people live with you in your home? *

Marcar apenas uma oval.

- 0
- 1
- 2
- 3
- 4 or more

10. How much was your income before the pandemic? Consider all the sources and all the people who live with you. *

Marcar apenas uma oval.

- 1-3 Minimum Wages
- 4-6 Minimum Wages
- More than 6 Minimum Wages

11. How much has the income in your home been impaired by the pandemic? Consider all the sources and all the people who live with you. *

Marcar apenas uma oval.

- My income was not impaired.
- It has decreased, but I still get more than half what I did before.
- It has decreased, now I get half of what I used to earn.
- It has decreased, now I get less than half of what I used to earn.
- I'm not getting anything.

12. Do you have any financial assistance during the pandemic? It is possible to tick more than one option. Check all that apply. *

Marque todas que se aplicam.

- [ ] Financial aid from family members.
- [ ] Doing some work at home to contribute to the income.
- [ ] Using reservations I had.
- [ ] Bank or financial loan.
- [ ] Financial assistance from the government.
- [ ] I didn't have my income impaired and, therefore, I don't need any assistance.

Social distancing

13. Has your routine changed since the stay at home recommendation started? *

Marcar apenas uma oval.

- ( ) Yes
- ( ) No

14. How many days have you not met face to face relatives or friends who do not live in your house? *

Marcar apenas uma oval.

- ( ) 0 days - I have been meeting them as usual, I am not staying in social distancing.
- ( ) 1-3 days
- ( ) 4-7 days
- ( ) 8-15 days
- ( ) More than 15 days

15. How much support have you received from your relatives? Choose from 1 to 5 the level of support. *

Marcar apenas uma oval.

	1	2	3	4	5	
none	◯	◯	◯	◯	◯	very much

16. Check the days that you have left your home in the last week. It is possible to select more than one option. Check all that apply. *

Marque todas que se aplicam.

- [ ] Sunday
- [ ] Monday
- [ ] Tuesday
- [ ] Wednesday
- [ ] Thursday
- [ ] Friday
- [ ] Saturday
- [ ] I haven't left home any day in the past week.

17. If you left home in the last week, what was the maximum time you stayed out? *

Marcar apenas uma oval.

- ◯ Up to 1 hour
- ◯ 2 to 3 hours
- ◯ 4 hours or more
- ◯ I haven't left home in the past week.

Lifestyle and Health

18. In the last 15 days, in general, how would you rank your health? *

Marcar apenas uma oval.

	1	2	3	4	5	
really bad	◯	◯	◯	◯	◯	excellent

19. Have you been more concerned about your hygiene habits during this period? *

Marcar apenas uma oval.

	1	2	3	4	5	
No, nothing	◯	◯	◯	◯	◯	Yes, a lot

20. Has social distancing changed your stool (evacuation) frequency? *

Marcar apenas uma oval.

- ◯ Yes, frequency increased
- ◯ Yes, frequency decreased
- ◯ Not modified

21. How was your sleep quality before social distancing? *

Marcar apenas uma oval.

	1	2	3	4	5	
too bad	◯	◯	◯	◯	◯	very good

22. How has been your sleep quality in the last 15 days? *

Marcar apenas uma oval.

	1	2	3	4	5	
too bad	◯	◯	◯	◯	◯	very good

23. How was your physical activity level before social distancing? *

Marcar apenas uma oval.

	1	2	3	4	5	
Little active	◯	◯	◯	◯	◯	Very active

24. How has been your physical activity level in the last 15 days? *

Marcar apenas uma oval.

	1	2	3	4	5	
Little active	◯	◯	◯	◯	◯	Very active

25. How physically tired you used to feel in your daily routine before social distancing? *

Marcar apenas uma oval.

	1	2	3	4	5	
Nothing	◯	◯	◯	◯	◯	Very tired

26. How physically tired have you felt in the last 15 days? *

Marcar apenas uma oval.

	1	2	3	4	5	
Nothing	○	○	○	○	○	Very tired

27. Do you have difficulties carrying out your daily activities at home? Respond considering the past 15 days. *

Marcar apenas uma oval.

	1	2	3	4	5	
No, nothing.	○	○	○	○	○	Yes, a lot.

28. Did you suffer any kind of fall in this period of social distancing? *

Marcar apenas uma oval.

- ○ Yes
- ○ No

29. How many hours of the day, in the current routine, have you been exposed to the sun? *

Marcar apenas uma oval.

- ○ 0
- ○ 1-3 hours
- ○ 4-6 hours
- ○ 7 hours or more

30. How many fruits or fruit juice did you consume per day? (respond considering the last 15 days) *

Marcar apenas uma oval.

- none
- 1
- 2
- 3
- 4
- 5 or more

31. How many vegetables did you consume per day? (respond considering the last 15 days) *

Marcar apenas uma oval.

- none
- 1
- 2
- 3
- 4
- 5 or more

32. How much meat, chicken, fish or egg did you consume per day? (respond considering the last 15 days) *

Marcar apenas uma oval.

- none
- 1
- 2
- 3
- 4
- 5 or more

33. How much milk, cheese or yogurt did you consume per day? (respond considering the last 15 days) *

Marcar apenas uma oval.

- none
- 1
- 2
- 3
- 4
- 5 or more

34. What is the closest point of your feeling in the last 15 days? *

Marcar apenas uma oval.

	1	2	3	4	5	
Discouraged						Excited

35. What is the closest point of your feeling in the last 15 days? *

Marcar apenas uma oval.

	1	2	3	4	5	
Sad						Cheerful

36. Do you believe that your spirituality has helped you at this time? *

Marcar apenas uma oval.

	1	2	3	4	5	
Never						Frequently

37. Which items most explain what you are experiencing right now? You can check all that apply. *

Marcar apenas uma oval.

- Bored
- Cozy
- Busy
- Good
- Cautious
- Careful
- Unpleasant
- Difficult
- Funny
- Hopeful
- Happy
- Weak
- Grateful
- Restless
- Insecure
- Disturbed
- Practical
- Bad
- Alone
- Sad

38. What are you missing with social distancing? You can check all that apply. *

Marcar apenas uma oval.

- Hugging people
- Shaking hands with acquaintances
- Walking and going wherever I want
- Talk to people
- Being with my family
- Talking with friends
- Shopping
- Parties
- Attending to Church
- Going to the gym or other physical activities
- Freedom
- Strolling
- Being able to choose what I want
- Going out
- Having fun
- Making decisions
- Working
- Tranquility
- Meeting people
- Travelling

Este conteúdo não foi criado nem aprovado pelo Google.

Google Formulários
